# Trap-Door Incision for Penetrating Thoracic Trauma: An Obsolete Approach?

**DOI:** 10.1155/2014/798242

**Published:** 2014-08-03

**Authors:** Ana Fabregues Olea, Leire Zarain Obrador, Dolores Perez-Diaz, Fernando Turégano Fuentes

**Affiliations:** General and Emergency Surgery, Gregorio Marañón University General Hospital (GMUGH), 28007 Madrid, Spain

## Abstract

Penetrating injuries to the subclavian vessels are uncommon and very severe lesions. They are difficult to expose and carry a high mortality. “Trap-door” incisions have lately been dismissed as too mutilating for the occasional victim of a penetrating thoracic trauma with massive bleeding difficult that is to expose. We present a case of severe bleeding from a stab wound in the left subclavicular area in a heavy-built patient where a “trap-door” incision proved inevitable to expose and repair the injury, and most probably saved his life.

## 1. Introduction

Trap-door incisions have lately been dismissed as too mutilating for the occasional victim of a penetrating thoracic trauma with massive bleeding and difficult exposure.

We present a case of severe bleeding from a stab wound in the left subclavicular area in a heavy-built patient where a trap-door incision proved inevitable to expose and repair the injury and most probably saved his life.

## 2. Patient, Methods, and Results

A 40-year-old male patient was brought to the trauma bay in the emergency department after being injured by a large stab wound below the midportion of the left clavicle. He was reported by the EMS as being cocaine addict. Severe external bleeding was temporarily controlled by manual compression during transfer to the hospital. On admission, he was tachycardic and very hypotensive (BP of 70/30 mmHg, HR of 132 bpm). He was intubated, and three Foley catheters were introduced through the wound, which failed to control the bleeding due to the large size of the wound. As the patient was in extremis, an emergency room thoracotomy (ERT) was done at the trauma bay which allowed for blind manual compression of the bleeding vessel against the apex of the thorax from within the thoracic cavity. This proved very successful in stopping the external bleeding. He was then rushed to the OR where the ERT exposure proved insufficient to assess and control the bleeding as this was mainly external, with very little bleeding into the pleural space. A long supra- and infraclavicular incision was done while keeping the manual compression at the apex from within the thorax. The clavicle was divided with a gigli saw. Due to the large muscle mass of the patient, this incision failed to expose the bleeding site. A decision was taken to split the sternum thus transforming the incision into a trap-door approach. Only then a large laceration of the subclavian vein was exposed. The vein was ligated, and the incision was closed. The whole procedure lasted for 2 hours. The patient remained unstable throughout the surgery. He received 10 liters of crystalloids and 12 units of packed cells, platelets, and fibrinogen, and his blood pressure was maintained with the aid of high doses of noradrenaline infusion.

He was then taken to the ICU and then back to the OR two hours later for persistent bleeding through the chest drains and thoracic wall incisions. The trap-door incision was reopened and the bleeding was found to be a combination of “surgical” hemorrhage (incomplete hemostasis) and coagulopathy. Careful hemostasis was performed this time on the subclavian muscles, thoracic wall, and pleura.

The patient had a protracted course in the ICU, where he stayed for 23 days. He required mechanical ventilation, platelets, coagulation factors, and vasopressors (high doses of noradrenaline) during the first postoperative days. He was also sedated because of a prolonged agitation which was attributed to his addiction to cocaine. He developed bilateral pulmonary atelectasis, left-sided pleural effusion, and ventilation-associated pneumonia, but no tubular necrosis. A tracheostomy was performed on the 14th day due to difficulty in weaning.

He was finally transferred to the general surgery ward where he made a steady recovery. As a striking and very uncommon sequel, he developed severe blindness from bilateral ischemic optic neuropathy which was attributed to hypotension and the use of vasopressors.

On followup, he is free of pain at the incision and with good cosmetic results, and his blindness persists, with only a mild recovery of his eyesight.

## 3. Discussion

Penetrating injuries to the subclavian and axillary vessels are uncommon and very severe lesions, with a difficult exposure and a high mortality [1, 2]. One in five patients is admitted with no signs of life or in extremis [2].

Preoperative hemorrhage control is occasionally possible with a balloon tamponade technique by means of Foley catheters, as in other body locations [2, 3]. This manoeuver proved unsuccessful in our patient due to the large width of the wound. The impending cardiac arrest from exsanguination prompted us to perform an ERT to temporarily control the bleeding from within the thoracic cavity.

A classical surgical approach option in left subclavian penetrating injuries has been the trap-door incision [4]. This has lately been dismissed by some authors, Demetriades et al. among them, who conclude that these incisions are more painful and associated with severe postoperative bleeding and respiratory complications [2]. They recommend a supra- and infraclavicular approach with division of the clavicle, with or without the addition of a median sternotomy. In their series of 79 patients with penetrating injuries to the subclavian and axillary vessels, 18 (23%) who were admitted with no signs of life or in extremis underwent an ERT without any survivors [2]. The ERT in our case was prompted by impending cardiac arrest from exsanguinations. Pressure from within the thorax most probably saved his life. This maneuver allowed us to proceed to the operating room where a clavicular incision was performed in order to gain definite control of the bleeding. This incision is often sufficient to control the bleeding but, as the series by Demetriades et al. shows, in almost 50% of cases, it has to be extended to include sternotomy or thoracotomy to allow exposure [2]. This has also been corroborated in a more recent series from Baltimore [5] and also in a small series dealing exclusively with venous injuries [6]. In patients, in whom an ERT is done in order to control the bleeding, performing a clavicular incision with an extension to the sternum practically transforms these incisions into a trap-door incision. An interesting approach advocated by trauma surgeons in Cali, Colombia, (personal communication) in patients crashing at the trauma bay, is the performance of the so-called “Aztec thoracotomy,” L-shape thoracotomy, or thoracosternocostochondrotomy [7]. It would allow the repair of the injured vessel through the initial ERT by means of a cephalad extension of the incision cutting on the cartilage side of three costochondral joints, resulting in a L-shape thoracotomy. This incision has been advocated as an emergency prehospital on-scene thoracotomy when there is lack of equipment while waiting for the arrival of emergency medical teams [7].

A venous repair can be seldom done, and ligation is the preferred procedure. Only a transient edema has been seen when this is performed [2]. Reconstruction of the clavicle is advised for a better functional and cosmetic outcome [8].

In isolated venous injuries, the mortality can rise up to 80%, significantly higher than when an arterial or combined lesion is present [9]. This has been explained on the basis of the absence of vasoconstriction, a higher amount of transfused blood (5 liters on average), and the risk of air embolism.

Our patient developed blindness. Ischemic optic anterior neuropathy has been previously described in victims of severe trauma. Risk factors for developing ischemic optic anterior neuropathy are severe hypotension, a sudden drop in hematocrit, aggressive fluid resuscitation, long term use of vasoactive amines, and the prone position. Except for this last one, our patient was exposed to all the other risk factors. Prognosis is poor, and only a few patients recover some visual acuity [10].

In conclusion, trap-door incisions are infrequently used nowadays for subclavian vessel injuries. Nevertheless, when the patient with thoracic injury at the trauma bay is crashing from near exsanguination, an ERT is unavoidable. Once temporary control is achieved, and the next logical incision is a clavicular one. This may fail to expose the injured vessel in the occasional athletic patient, making it inevitable to extend the incision by performing a median sternotomy. This uncommon approach should be kept in mind in order to save patients' lives.

## References

[1] Kessel B, Ashkenazi I, Portnoy I, Hebron D, Eilam D, Alfici R (2009). Right-sided “trapdoor” incision provides necessary exposure of complex cervicothoracic vascular injury. *Scandinavian Journal of Trauma, Resuscitation and Emergency Medicine*.

[2] Demetriades D, Chahwan S, Gomez H (1999). Penetrating injuries to the subclavian and axillary vessels. *Journal of the American College of Surgeons*.

[3] Gilroy D, Lakhoo M, Charalambides D, Demetriades D (1992). Control of life-threatening haemorrhage from the neck: a new indication for balloon tamponade. *Injury*.

[4] Demetriades D, Asensio JA, Velmahos G, Thal E (1996). Complex problems in penetrating neck trauma. *Surgical Clinics of North America*.

[5] O'Connor JV, Scalea TM (2010). Penetrating thoracic great vessel injury: Impact of admission hemodynamics and preoperative imaging. *The Journal of Trauma*.

[6] Baumgartner FJ, Rayhanabad J, Bongard FS, Milliken JC, Donayre C, Klein SR (1999). Central venous injuries of the subclavian-jugular and innominate-caval confluences. *Texas Heart Institute Journal*.

[7] Ashrafian H, Athanasiou T (2010). Emergency prehospital on-scene thoracotomy: a novel method. *Collegium Antropologicum*.

[8] Old W, Oswaks R (1982). Clavicular excision in management of vascular trauma. * The American Surgeon*.

[9] Demetriades D, Rabinowitz B, Pezikis A, Franklin J, Palexas G (1987). Subclavian vascular injuries. *The British Journal of Surgery*.

[10] Raigal Caño A, Hortigüela Martín V, Sánchez Carretero MJ, Sánchez Casado M, de Toro Martín-Consuegra IL, Marina Martínez L (2008). Neuropatía óptica isquémica en el paciente politraumatizado. *Medicina Intensiva*.

